# Allium vegetable consumption and health: An umbrella review of meta‐analyses of multiple health outcomes

**DOI:** 10.1002/fsn3.1117

**Published:** 2019-07-10

**Authors:** Qianyi Wan, Ni Li, Liang Du, Rui Zhao, Mengshi Yi, Qiushi Xu, Yong Zhou

**Affiliations:** ^1^ Department of Gastrointestinal Surgery, West China Hospital Sichuan University Chengdu China; ^2^ Department of Ophthalmology, West China Hospital Sichuan University Chengdu China; ^3^ Chinese Evidence‐Based Medicine/Cochrane Center Chengdu China; ^4^ West China School of Medicine Sichuan University Chengdu China

**Keywords:** allium vegetables, garlic, health‐related outcomes, onion, umbrella review

## Abstract

Previously, our meta‐analysis and other studies have suggested that allium vegetable consumption is beneficial for health, but no umbrella review has been conducted to assess the evidence of the various health benefits of allium vegetable consumption. Therefore, we conducted this umbrella review on this topic. This umbrella review included a total of 16 meta‐analyses with 50 unique outcomes. The most beneficial cancer‐related outcome was shown for gastric cancer (risk ratio 0.78; 95% confidence interval [CI] 0.67–0.91). In addition, only 8 weeks of garlic consumption significantly decreased serum total cholesterol (weighted mean differences −17.20 mg/dl; 95% CI −23.10 to −11.30), and patients with dyslipidemia who consumed garlic experienced more benefits than the whole population. Diabetic patients with longer durations of garlic intake experienced more benefits in terms of fasting blood glucose (FBG), HbA1c, and serum fructosamine than healthy participants, and garlic intake was associated with blood pressure reduction in hypertensive patients but not in normotensive participants. Limited side effects of garlic, such as garlic odor and gastrointestinal complaints, were reported among the included meta‐analyses. Our results suggested that allium vegetables might be beneficial for cancer prevention. In particular, garlic was comparatively safe and is recommended as a long‐term dietary component for patients with dyslipidemia, diabetes, and hypertension.

## 1 INTRODUCTION

Allium vegetables, such as garlic and onion, are widely used herbal supplements (Morris & Avorn, 2003). For example, it was estimated that the worldwide onion production is approximately 78.31 million tons (Teshika et al., 2018). For a long time, allium vegetables have been used as foods and spices in many countries, and they have also been used in most cultures for various medicinal purposes (Pittler & Ernst, 2007).

Previously, we performed a meta‐analysis and found that the consumption of large amounts of allium vegetables, such as onion, garlic, leek, and Chinese chive, could reduce the risk of gastric cancer (Zhou et al., 2011). In addition to gastric cancer, further studies also suggested that onion and garlic were beneficial for the prevention of multiple cancers, such as laryngeal and esophageal cancer (Galeone et al., 2006). We also identified other studies that focused on the effects of allium vegetable consumption on other health‐related outcomes. Clinical trials have suggested that garlic supplementation could reduce atherogenic markers and thus may have a cardioprotective effect (Jung et al., 2014), and garlic was also recommended as a nutritional supplement to prevent cardiovascular disease (Eilat‐Adar, Sinai, Yosefy, & Henkin, 2013). Moreover, it was indicated that aged garlic extract had a blood pressure‐lowering effect and thus might be a safe adjunct treatment in addition to conventional antihypertensive therapy (Ried, Frank, & Stocks, 2013).

The benefits of allium vegetable consumption have been reported by many studies; however, contradictory results still exist. There were inconsistencies among several meta‐analyses about the associations between allium vegetable consumption and cancer, metabolic outcomes, and other health‐related outcomes (Chiavarini, Minelli, & Fabiani, 2016; Sahebkar, Serban, Ursoniu, & Banach, 2016; Shabani, Sayemiri, & Mohammadpour, 2018; Turati, Guercio, Pelucchi, La Vecchia, & Galeone, 2014), and these differences might have resulted from the different participants, types of allium vegetables, duration of allium vegetable consumption, and other factors in each meta‐analysis. Recently, an umbrella review was published and reported that garlic had some positive effects on indicators and biomarkers of cardiovascular disease; however, other types of allium vegetables and other health‐related outcomes were not mentioned (Schwingshackl, Missbach, & Hoffmann, 2016). Considering that previous studies that assessed the medical effects of allium vegetables were restricted to a single kind of health‐related outcome, no study has systematically evaluated the associations between allium vegetables and multiple health‐related outcomes. To better understand this issue, we systematically searched for relevant articles and performed this umbrella review.

## 2 MATERIALS AND METHODS

An umbrella review is a review of existing systematic reviews and/or meta‐analyses (Aromataris et al., 2015). We conducted this umbrella review of allium vegetable consumption and multiple health‐related outcomes according to the standardized procedures described previously (Aromataris et al., 2015; Ioannidis, 2009).

### 2.1 Literature search and eligibility criteria

We searched PubMed, Embase, the Web of Science, and the Cochrane Library for relevant studies from the inception of the databases to December 2018. The following terms were used for the search: (allium* OR garlic* OR onion* OR chive* OR shallot* OR leek*) AND (systematic review* OR meta‐analysis*), and the terms were truncated for all fields (detailed search strategies are shown in the Supporting Information). The references of related studies were also reviewed to identify any meta‐analyses that were possibly missed in the initial search. Two researchers reviewed the identified studies independently to determine whether they were eligible for inclusion. The inclusion criteria were as follows: (a) The study was a meta‐analysis or a systematic review and meta‐analysis; (b) the study assessed the associations between any type of allium vegetable intake and health‐related outcomes in human subjects; and (c) the summary risk ratio (RR), odds ratio (OR), and hazard ratio for meta‐analyses of observational studies or the summary mean differences for meta‐analyses of interventional studies were reported. Only studies published in English were included. Systematic reviews without meta‐analyses and animal studies were excluded from this umbrella review. All differences were discussed by two researchers, and disagreements were resolved by consensus.

### 2.2 Data extraction

Two researchers extracted the data independently, and the information that was extracted was as follows: name of first author; publication year; health‐related outcomes; type, dose, and duration of allium vegetable consumption; characteristics of the study population; type and number of studies or trials; number of participants; metrics; summary estimates; and related 95% confidence intervals (CIs). When a meta‐analysis investigated more than one health‐related outcome, we extracted each outcome separately. If a meta‐analysis investigated a health‐related outcome with several types of allium vegetables and different populations, we would extract the outcome with a single type of allium vegetable and a specific population separately. We regarded each outcome as an independent outcome and selected the most recent meta‐analysis for the data analysis. If more than one meta‐analysis was performed within the same 2‐year period for the same outcome, we selected the one that included the largest number of studies.

### 2.3 Assessment of methodological quality and quality of evidence of the included meta‐analyses

The methodological quality of each included study was assessed according to a measurement tool to assess systematic reviews (AMSTAR), which was a measurement tool consisting of 11 items that has been shown to have good agreement, reliability, construct validity, and feasibility for assessing systematic reviews (Shea et al., 2007, 2009). We also used the Grading of Recommendations Assessment, Development, and Evaluation (GRADE) system to assess the quality of the evidence of the included studies. GRADE is an approach that offers a transparent and structured process for developing and presenting summaries of evidence (Guyatt et al., 2011). In GRADE, the quality of the evidence was divided into four categories (high, moderate, low, and very low), and randomized trials had a higher quality of evidence than observational studies.

### 2.4 Data analysis

We extracted only each health‐related outcome reported in the meta‐analysis instead of reanalyzing the summary estimates and 95% CIs, and we did not search for the primary studies included in the meta‐analysis. For each health‐related outcome, if a related meta‐analysis was performed with both a random effects model and a fixed effects model, we primarily chose the one with the random effects model as the final outcome. When the value of the *I^2^* metric and test of publication bias were included in related meta‐analysis, we extracted them as the measures of heterogeneity and publication bias. If the values were not included, we would calculate the *I^2^* statistic to assess heterogeneity, and we also performed the Egger's regression test for the health‐related outcome in related meta‐analysis that included at least 10 studies to assess the publication bias if the detailed original data were available (Egger, Davey Smith, Schneider, & Minder, 1997; Higgins, Thompson, Deeks, & Altman, 2003). *I^2^* >50% was regarded as substantial heterogeneity, and a *p* value of <0.1 for Egger's regression test indicated statistically significant publication bias.

## 3 RESULTS

**Figure 1:**
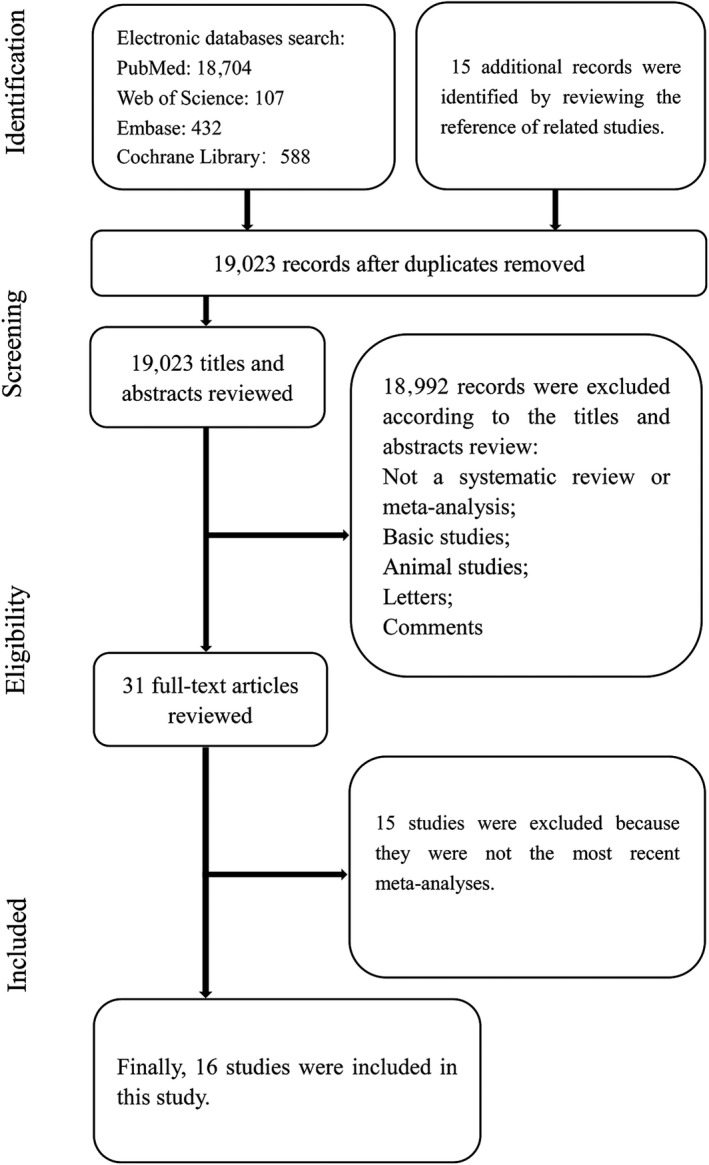
Flow chart of the selection process

Figure 1 shows a detailed flowchart of the selection process. Through the initial search, we identified 18,704 articles from PubMed, 107 articles from the Web of Science, 432 articles from Embase, and 588 articles from the Cochrane Library. Additionally, 15 articles were identified by reviewing the reference of the related studies. Then, 19,023 articles remained after removing the duplicates, and 18,992 articles were excluded after reviewing the titles and abstracts. Finally, 31 full‐text articles (Chiavarini et al., 2016; Emami, Rouhani, & Azadbakht, 2017; Fleischauer, Poole, & Arab, 2000; Guercio, Turati, La Vecchia, Galeone, & Tavani, 2016; Hu et al., 2014; Khoo & Aziz, 2009; Kodali & Eslick, 2015; Kwak et al., 2014; Li, Ying, Shan, & Ji, 2018; Reinhart, Coleman, Teevan, Vachhani, & White, 2008; Reinhart, Talati, White, & Coleman, 2009; Ried, 2016; Ried, Frank, Stocks, Fakler, & Sullivan, 2008; Ried, Toben, & Fakler, 2013; Rohner, Ried, Sobenin, Bucher, & Nordmann, 2015; Sahebkar et al., 2016; Shabani et al., 2018; Silagy & Neil, 1994; Stevinson, Pittler, & Ernst, 2000; Sun, Wang, & Qin, 2018; Taghizadeh, Hamedifard, & Jafarnejad, 2018; Turati et al., 2014; Turati, Pelucchi, Guercio, La Vecchia, & Galeone, 2015; Wang, Yang, Qin, & Yang, 2015; Wang, Zhang, Lan, & Wang, 2017; Warshafsky, Kamer, & Sivak, 1993; Xiong et al., 2015; Zeng et al., 2012; Zhou, Ding, & Liu, 2013; Zhou et al., 2011; Zhu, Zou, Qi, Zhong, & Miao, 2014) were reviewed for further assessment.

**Figure 2:**
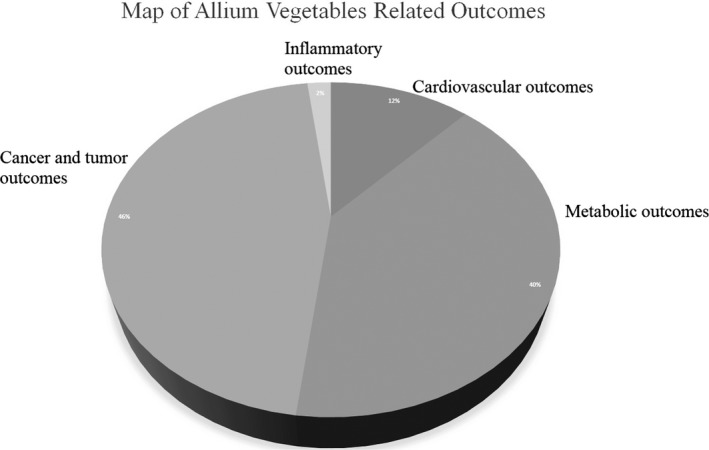
The map of allium vegetable‐related outcomes

Among the 31 studies, 11 studies (Chiavarini et al., 2016; Fleischauer et al., 2000; Guercio et al., 2016; Hu et al., 2014; Kodali & Eslick, 2015; Li et al., 2018; Turati et al., 2014, 2015; Zhou et al., 2013, 2011; Zhu et al., 2014) were about cancer and tumor outcomes (gastric cancer, colorectal cancer, upper aerodigestive tract cancer, prostate cancer, and colorectal adenomatous polyps), 13 studies (Emami et al., 2017; Khoo & Aziz, 2009; Kwak et al., 2014; Reinhart et al., 2009; Ried, 2016; Ried, Toben, et al., 2013; Sahebkar et al., 2016; Shabani et al., 2018; Stevinson et al., 2000; Sun et al., 2018; Wang et al., 2017; Warshafsky et al., 1993; Zeng et al., 2012) were about metabolic outcomes (serum total cholesterol [TC], high‐density lipoprotein [HDL], low‐density lipoprotein [LDL], triglycerides [TGs], fasting blood glucose [FBG], serum HbA1c, serum fructosamine, serum lipoprotein (a), and apolipoprotein B), seven studies (Reinhart et al., 2008; Ried, 2016; Ried et al., 2008; Rohner et al., 2015; Silagy & Neil, 1994; Wang et al., 2015; Xiong et al., 2015) were about cardiovascular outcomes (systolic blood pressure and diastolic blood pressure), and only one systematic review and meta‐analysis (Taghizadeh et al., 2018) was about serum C‐reactive protein levels. Finally, 50 unique outcomes extracted from the 16 most recent meta‐analyses were analyzed in this umbrella review, and the map of allium vegetable‐related outcomes is reported in Figure 2.

### 3.1 Cancer and tumor outcomes

A total of 23 outcomes, involving seven kinds of cancers and tumors and four types of allium vegetables, were reported in seven (Chiavarini et al., 2016; Guercio et al., 2016; Li et al., 2018; Turati et al., 2014, 2015; Zhou et al., 2013; Zhu et al., 2014) meta‐analyses (Table 1). Among these meta‐analyses, the median number of studies was 6 (range 3–18) and the median number of participants was 91,368 (range 1,595–552,180).

**Table 1:** Associations between allium vegetables consumption and cancer and tumor outcomes

Outcome	Author	Year	Type of allium vegetables	Population	Type of studies in MA	No. of studies and participants in MA	Metric of MA	Effects model	Effect size (95% CI)	*I* ^2^%^a^	Publication bias^b^
Gastric cancer	Li, Z. Y., et al	2018	Garlic or garlic powder	Participants from Asia, Europe, and America	1 interventional study, 2 cohort, and 15 case–control studies	18; 142,921	OR	REM	0.51 (0.44, 0.57)	59.6	0.9
Gastric cancer	Turati, F., et al	2015	Onion	Participants from Asia, Europe, and South America	13 case–control studies	13; 4,619	RR	REM	0.55 (0.41, 0.73)	76.0	0.04
Gastric cancer	Turati, F., et al	2015	Chinese chives	Participants from Asia, Europe, and South America	3 case–control studies	3; 1,595	RR	REM	0.43 (0.26, 0.73)	70.9	None
Gastric cancer	Turati, F., et al	2015	Allium vegetables	Participants from Asia, Europe, and South America	4 cohort and 10 case–control studies	14; 6,227	RR	REM	0.78 (0.67, 0.91)	58.6	None
Colorectal cancer	Chiavarini, M., et al	2016	Garlic or garlic supplement	Participants from China, Europe, Argentina and America	7 cohort and 7 case–control studies	14; 535,901	OR	REM	0.93 (0.82, 1.06)	83.6	0.121
Colorectal cancer	Turati, F., et al	2014	Onion	Participants from Asia, Europe, Australia, and North and South America	2 cohort and 4 case–control studies	6; NR	RR	REM	0.85 (0.7, 1.04)	46.1	0.106
Colorectal cancer	Zhu, B. B., et al	2014	Allium vegetables	Participants from China, the Netherlands, and America	Observational studies	8; 552,180	RR	FEM	1.06 (0.96, 1.17)	0	0.35
Colorectal cancer of male	Chiavarini, M., et al	2016	Garlic or garlic supplement	Participants from China, Europe, Argentina, and America	3 cohort and 1 case–control studies	4; 141,341	OR	REM	1.02 (0.89, 1.18)	0	0.848
Colorectal cancer of female	Chiavarini, M., et al	2016	Garlic or garlic supplement	Participants from China, Europe, Argentina, and America	3 cohort and 2 case–control studies	5; 115,784	OR	REM	0.94 (0.79, 1.12)	54.9	0.005
Colon cancer	Chiavarini, M., et al	2016	Garlic or garlic supplement	Participants from China, Europe, Argentina, and America	5 cohort and 1 case–control studies	6; 200,711	OR	REM	0.93 (0.74, 1.16)	71.1	0.026
Rectal cancer	Chiavarini, M., et al	2016	Garlic or garlic supplement	Participants from China, Europe, Argentina, and America	2 cohort and 1 case–control studies	3; 91,368	OR	REM	1.00 (0.69, 1.45)	41.4	0.061
Colorectal adenomatous polyps	Turati, F., et al	2014	Allium vegetables	Participants from America	Case–control studies	3; NR	RR	FEM	0.88 (0.80, 0.98)	0	0.756
Upper aerodigestive tract cancer	Guercio, V., et al	2016	Allium vegetables	Worldwide countries	3 cohort and 4 case–control studies	7; 368,435	RR	REM	0.79 (0.56, 1.11)	76.5	None
Upper aerodigestive tract cancer	Guercio, V., et al	2016	Garlic	Worldwide countries	10 case–control studies	10; 22,660	RR	REM	0.74 (0.57, 0.95)	76.9	None
Upper aerodigestive tract cancer	Guercio, V., et al	2016	Onion	Worldwide countries	1 cohort and 7 case–control studies	8; 19,229	RR	REM	0.72 (0.57, 0.91)	60.6	None
Esophagus cancer	Guercio, V., et al	2016	Allium vegetables	Worldwide countries	Observational studies	4; NR	RR	REM	0.65 (0.31, 1.35)	NR	NR
Esophagus cancer	Guercio, V., et al	2016	Garlic	Worldwide countries	Observational studies	8; NR	RR	REM	0.68 (0.50, 0.92)	NR	NR
Esophagus cancer	Guercio, V., et al	2016	Onion	Worldwide countries	Observational studies	5; NR	RR	REM	0.66 (0.45, 0.97)	NR	NR
Head and neck cancer	Guercio, V., et al	2016	Garlic	Worldwide countries	Observational studies	3; NR	RR	REM	0.95 (0.57, 1.57)	NR	NR
Head and neck cancer	Guercio, V., et al	2016	Onion	Worldwide countries	Observational studies	3; NR	RR	REM	0.78 (0.57, 1.05)	NR	NR
Larynx cancer	Guercio, V., et al	2016	Onion	Worldwide countries	Observational studies	3; NR	RR	REM	0.72 (0.53, 0.97)	NR	NR
Prostate cancer	Zhou, X. F., et al	2013	Garlic	Participants from Asia, Europe, Australia, and America	2 cohort and 5 case–control studies	7; 68,600	OR	REM	0.77 (0.64, 0.91)	63.6	0.015
Prostate cancer	Zhou, X. F., et al	2013	Onion	Participants from Asia, Europe, Australia, and America	1 cohort and 3 case–control studies	4; 33,470	OR	FEM	0.84 (0.62, 1.13)	49.4	0.015

Note

95% CI, 95% confidence intervals; FEM, fixed effects model; NR, not reported; MA, meta‐analysis; OR, odds ratios; REM, random effects model; RR, relative risk.

a Heterogeneity was assessed with *I* ^2^ statistic.

b Assessed by Egger's regression test.

Among the cancers and tumors, the greatest benefits of allium vegetable consumption were shown for gastric cancer. For the associations between gastric cancer risk and Chinese chives, garlic, onion, and allium vegetable consumption, the summary results were RR 0.43 (95% CI 0.26–0.73), OR 0.51 (95% CI 0.44–0.57), RR 0.55 (95% CI 0.41–0.73), and RR 0.78 (95% CI 0.67–0.91), respectively. For colorectal cancer, the results from three meta‐analyses (Chiavarini et al., 2016; Turati et al., 2014; Zhu et al., 2014) showed that garlic, onion, and allium vegetable consumption had no significant associations with the risk of colorectal cancer. Stratification of colon cancer, rectal cancer, and colorectal cancer in males and females also showed no significant associations. A single meta‐analysis (Turati et al., 2014) suggested that allium vegetables could reduce the risk of colorectal adenomatous polyps (RR 0.88; 95% CI 0.80–0.98). For prostate cancer, the protective effect was shown only for garlic consumption (OR 0.77; 95% CI 0.64–0.91), while onion had no significant effect.

For the risk of upper aerodigestive tract cancer, both garlic and onion, but not all allium vegetables, showed significant beneficial effects. After stratification by subsite of cancer, inverse associations were observed between garlic and the risk of esophageal cancer, onion and the risk of esophageal cancer, and onion and the risk of laryngeal cancer. No significant associations were observed between allium vegetables and esophageal cancer or between garlic and onion and the risk of head and neck cancer (Table 1).

### 3.2 Metabolic outcomes

A total of 12 studies that reported the associations between garlic consumption and metabolic outcomes were included in this umbrella review, and we analyzed the 20 metabolic outcomes in different populations that were reported in the six most recent meta‐analyses (Emami et al., 2017; Ried, Toben, et al., 2013; Sahebkar et al., 2016; Shabani et al., 2018; Wang et al., 2017; Zeng et al., 2012). All six meta‐analyses of metabolic outcomes included only interventional studies, and of the only allium vegetables investigated were garlic‐ and garlic‐related supplements. Among the six meta‐analyses, the median daily dose of garlic was 900 mg/day (range 890–1,700 mg/day), and the median duration of the studies was 12 weeks (range 6–12 weeks). Among the 20 metabolic outcomes, the median number of trials was 10.5 (range 2–37). Most of the outcomes were analyzed by using the random effects model, while only one outcome was analyzed using a fixed effects model.

Table 2 reports detailed information about the 20 metabolic outcomes. The results showed that participants (including both healthy participants and patients with dyslipidemia) who consumed garlic had reduced serum levels of TC (weighted mean differences [WMD] −15.25 mg/dl; 95% CI −20.72 to −9.78) and LDL (WMD −6.41 mg/dl; 95% CI −11.77 to −1.05) and increased serum levels of HDL (WMD 1.49 mg/dl; 95% CI 0.19–2.79). No statistically significant change was observed in the serum levels of TGs (WMD −5.45 mg/dl; 95% CI −14.18–3.27). Garlic intake resulted in greater reductions in serum TC (WMD −16.87 mg/dl; 95% CI −21.01 to −12.73) and LDL (WMD −9.65 mg/dl; 95% CI −15.07 to −4.23) and greater increases in serum of HDL (WMD 3.19 mg/dl; 95% CI 1.85–4.53) only patients with dyslipidemia, and garlic intake also showed a decreasing effect on serum TGs (WMD −12.44 mg/dl; 95% CI −18.19 to −6.69). For participants with baseline TC levels ≤200 mg/dl, garlic intake did not show a statistically significant change in serum TC (WMD −5.73 mg/dl; 95% CI −14.31–2.85). The results of stratification by duration of garlic intake showed that a short duration (2–8 weeks) resulted in no statistically significant change in serum TC, while a long duration (>8 weeks) of garlic intake significantly decreased serum TC (WMD −17.20 mg/dl; 95% CI −23.10 to −11.30).

**Table 2:** Associations between garlic consumption and metabolic outcomes

Outcome	Author	Year	Type of allium vegetables	Dose	Population	Type of studies in MA	No. Of trials and participants in MA	Duration	Metric of MA	Units	Effects model	Effect size (95% CI)	*I* ^2^%^a^	Publication bias
Total cholesterol	Shabani, E., et al	2018	Garlic	Median: 1,000 mg/day (range 80–20,000)	Patients with dyslipidemia	Interventional studies	31; NA	Median: 80.5 days (range 2–180)	WMD	mg/dl	REM	−16.87 (−21.01, −12.73)	92.2	Low risk^b^
Total cholesterol^c^	Ried, K., et al	2013	Garlic (powder, oil, garlic extract, or raw garlic)	Median: 900 mg/day (range 4–22,400)	Both healthy participants and patients with dyslipidemia	Interventional studies	37; NA	Median: 12 weeks (range 2–52)	WMD	mg/dl	REM	−15.25 (−20.72, −9.78)	77.0	None^d^
Total cholesterol	Ried, K., et al	2013	Garlic (powder, oil, garlic extract, or raw garlic)	Median: 900 mg/day (range 4–22,400)	Participants with short duration: 2–8 weeks	Interventional studies	6; NA	Median: 12 weeks (range 2–52)	WMD	mg/dl	REM	−1.59 (−12.45, 9.27)	0	NR
Total cholesterol	Ried, K., et al	2013	Garlic (powder, oil, garlic extract, or raw garlic)	Median: 900 mg/day (range 4–22,400)	Participants with long duration: >8 weeks	Interventional studies	31; NA	Median: 12 weeks (range 2–52)	WMD	mg/dl	REM	−17.20 (−23.10, −11.30)	79.0	NR
Total cholesterol	Ried, K., et al	2013	Garlic (powder, oil, garlic extract, or raw garlic)	Median: 900 mg/day (range 4–22,400)	Participants with TC baseline ≤200 mg/dl	Interventional studies	8; NA	Median: 12 weeks (range 2–52)	WMD	mg/dl	REM	−5.73 (−14.31, 2.85)	0	NR
HDL	Shabani, E., et al	2018	Garlic	Median: 1,000 mg/day (range 80–20,000)	Patients with dyslipidemia	Interventional studies	29; NA	Median: 80.5 days (range 2–180)	WMD	mg/dl	REM	3.19 (1.85, 4.53)	93.0	Low risk^b^
LDL	Shabani, E., et al	2018	Garlic	Median: 1,000 mg/day (range 80–20,000)	Patients with dyslipidemia	Interventional studies	28; NA	Median: 80.5 days (range 2–180)	WMD	mg/dl	REM	−9.65 (−15.07, −4.23)	96.2	Low risk^b^
Triglycerides	Shabani, E., et al	2018	Garlic	Median: 1,000 mg/day (range 80–20,000)	Patients with dyslipidemia	Interventional studies	28; NA	Median: 80.5 days (range 2–180)	WMD	mg/dl	REM	−12.44 (−18.19, −6.69)	93.6	Low risk^b^
HDL^c^	Ried, K., et al	2013	Garlic (powder, oil, garlic extract, or raw garlic)	Median: 900 mg/day (range 4–22,400)	Both healthy participants and patients with dyslipidemia	Interventional studies	30; NA	Median: 12 weeks (range 2–52)	WMD	mg/dl	REM	1.49 (0.19, 2.79)	33.0	None^d^
LDL^c^	Ried, K., et al	2013	Garlic (powder, oil, garlic extract, or raw garlic)	Median: 900 mg/day (range 4–22,400)	Both healthy participants and patients with dyslipidemia	Interventional studies	26; NA	Median: 12 weeks (range 2–52)	WMD	mg/dl	REM	−6.41 (−11.77, −1.05)	75.0	None^d^
Triglycerides^c^	Ried, K., et al	2013	Garlic (powder, oil, garlic extract, or raw garlic)	Median: 900 mg/day (range 4–22,400)	Both healthy participants and patients with dyslipidemia	Interventional studies	32; NA	Median: 12 weeks (range 2–52)	WMD	mg/dl	REM	−5.45 (−14.18, 3.27)	71.0	None^d^
Fasting blood glucose ( FBG)	Shabani, E., et al	2018	Garlic	Median: 1,000 mg/day (range 80–20,000)	Diabetic patients	Interventional studies	13; NA	Median: 80.5 days (range 2–180)	WMD	mg/dl	REM	−10.90 (−16.40, −5.40)	94.9	None^b^
FBG	Emami, S., et al	2017	Garlic (garlic food or supplement)	Median: 1,700 mg/day (range 500–40,000)	Healthy participants	Interventional studies	3; NA	Median: 42 days (range 2–168)	WMD	mg/dl	REM	−0.68 (−6.16, 4.81)	21.1	None^d^
HbA1c	Shabani, E., et al	2018	Garlic	Median: 1,000 mg/day (range 80–20,000)	Diabetic patients	Interventional studies	7; NA	Median: 80.5 days (range 2–180)	WMD	mg/dl	REM	−0.60 (−0.98, −0.22)	93.2	Low risk^b^
HbA1c	Wang, J., et al	2017	Garlic supplement	Median: 900 mg/day (range 50–2,400)	Type 2 diabetic patients	Interventional studies	2; 360	12 weeks	SMD	NR	REM	−6.93 (−10.71, −3.14)	98.0	Moderate risk^b^
HbA1c	Wang, J., et al	2017	Garlic supplement	Median: 900 mg/day (range 50–2,400)	Type 2 diabetic patients	Interventional studies	2; 300	24 weeks	SMD	NR	REM	−13.25 (−15.83, −10.68)	82.0	Moderate risk^b^
Serum fructosamine	Wang, J., et al	2017	Garlic supplement	Median: 900 mg/day (range 50–2,400)	Type 2 diabetic patients	Interventional studies	2; 120	1–2 weeks	SMD	NR	REM	−1.92 (−2.85, −0.99)	75.0	None^b^
Serum fructosamine	Wang, J., et al	2017	Garlic supplement	Median: 900 mg/day (range 50–2,400)	Type 2 diabetic patients	Interventional studies	2; 172	3–4 weeks	SMD	NR	REM	−3.48 (−6.25, −0.71)	96.0	None^b^
Lipoprotein (a)	Sahebkar, A., et al	2016	Garlic supplement	Median: 900 mg/day (range 250–900)	Patients with dyslipidemia	Interventional studies	6; 256	Median: 12 weeks (range 8–52)	WMD	mg/dl	REM	16.86 (−4.59, 38.31)	NR	0.08^e^
Apolipoprotein B	Zeng, T., et al	2012	Garlic	Median: 890 mg/day (range 4–600,000)	Both healthy and hypercholesterolemic participants	Interventional studies	4; 112	Median: 12 weeks (range 2–48)	WMD	mg/ml	FEM	−0.03 (−0.13, 0.08)	25.6	0.702^e^

Note

95% CI, 95% confidence intervals; FEM, fixed effects model; MA, meta‐analysis; NA, not available; NR, not reported; REM, random effects model; SMD, standard mean differences; WMD, weighted mean differences.

a Heterogeneity was assessed with *I* ^2^ statistic.

b Assessed by funnel chart.

c Patients that requiring cholesterol‐lowering medical treatments were excluded.

d Assessed by Begg's funnel plots.

e Assessed by Egger's regression test.

For diabetic patients, garlic lowered the levels of FBG (WMD −10.90 mg/dl; 95% CI −16.40 to −5.40) and HbA1c (WMD −0.60 mg/dl; 95% CI −0.98 to −0.22), but no significant associations were shown between garlic intake and the FBG of healthy participants. In type 2 diabetic patients who underwent two different durations of garlic supplementation, both the 12‐week duration (standard mean differences [SMD] −6.93; 95% CI −10.71 to −3.14) and the 24‐week duration (SMD −13.25; 95% CI −15.83 to −10.68) of garlic supplementation significantly reduced the level of HbA1c, and this effect was greater in the patients who consumed garlic for a duration of 24 weeks than in those who consumed garlic for 12 weeks. We also found that garlic significantly decreased the serum fructosamine level of type 2 diabetic patients after consuming garlic for a duration of “1–2 weeks,” as well as a duration of “3–4 weeks.” No significant associations were found between garlic consumption and serum lipoprotein (a) and apolipoprotein B levels.

### 3.3 Cardiovascular outcomes

The associations between garlic consumption and blood pressure in different populations were reported with six outcomes in the two most recent meta‐analyses (Ried, 2016; Rohner et al., 2015) (Table 3). The median number of trials included in these meta‐analyses was 12.5 (range 9–20), and the median number of participants was 561.5 (range 468–972). In the two meta‐analyses, the median daily doses of garlic were 600 and 900 mg/d, and the median duration was 12 weeks in both meta‐analyses. All the analyses of the six outcomes were performed with a random effects model.

**Table 3:** Associations between garlic consumption and cardiovascular and inflammatory outcomes

Outcome	Author	Year	Type of allium vegetables	Dose	Population	Type of studies in MA	No. Of trials and participants in MA	Duration	Metric of MA	Units	Effects model	Effect size (95% CI)	*I* ^2^%^a^	Publication bias
Systolic blood pressure	Ried, K.	2016	Garlic (powder, extract, or oil)	Median: 900 mg/day (range 12.3–3,050)	Both normotensive and hypertensive participants	Interventional studies	19; 908	Median: 12 weeks (range 2–24)	WMD	mmHg	REM	−5.07 (−7.30, −2.85)	71.0	NR
Diastolic blood pressure	Ried, K.	2016	Garlic (powder, extract, or oil)	Median: 900 mg/day (range 12.3–3,050)	Both normotensive and hypertensive participants	Interventional studies	20; 972	Median: 12 weeks (range 2–24)	WMD	mmHg	REM	−2.48 (−4.07, −0.89)	72.0	NR
Systolic blood pressure	Ried, K.	2016	Garlic (powder, extract, or oil)	Median: 900 mg/day (range 12.3–3,050)	Hypertensive participants	Interventional studies	10; 468	Median: 12 weeks (range 2–24)	WMD	mmHg	REM	−8.35 (−10.58, −6.11)	48.0	NR
Diastolic blood pressure	Rohner, A., et al	2015	Garlic (powder, extract)	Median: 600 mg/day (range 188–960)	Hypertensive participants	Interventional studies	9; 482	Median: 12 weeks (range 8–26)	WMD	mmHg	REM	−3.82 (−6.69, −0.96)	80.0	None^b^
Systolic blood pressure	Ried, K.	2016	Garlic (powder, extract, or oil)	Median: 900 mg/day (range 12.3–3,050)	Normotensive participants	Interventional studies	11; 468	Median: 12 weeks (range 2–24)	WMD	mmHg	REM	−1.50 (−3.40, 0.40)	NR	NR
Diastolic blood pressure	Ried, K.	2016	Garlic (powder, extract, or oil)	Median: 900 mg/day (range 12.3–3,050)	Normotensive participants	Interventional studies	14; 641	Median: 12 weeks (range 2–24)	WMD	mmHg	REM	−0.40 (−1.60, 0.80)	NR	NR
C‐reactive protein level	Taghizadeh, M., et al	2018	Garlic and garlic supplement	Median: 2,100 mg/day (range 250–3,600)	Healthy participants or patients of diabetes and coronary artery disease	Interventional studies	9; 348	Median: 12 weeks (range 2–48)	WMD	mg/L	REM	−0.80 (−1.50, −0.10)	98.0	0.34^c^

Note

95% CI, 95% confidence intervals; MA, meta‐analysis; NR, not reported; REM, random effects model; WMD, weighted mean differences.

a Heterogeneity was assessed with *I* ^2^ statistic.

b Assessed by funnel chart.

c Assessed by Egger's regression test.

The summary results for all participants showed that garlic could significantly lower systolic blood pressure (WMD −5.07 mmHg; 95% CI −7.30 to −2.85) and diastolic blood pressure (WMD −2.48 mmHg; 95% CI −4.07 to −0.89). After stratification by population, the results showed that garlic had a larger effect on lowering both systolic blood pressure (WMD −8.35 mmHg; 95% CI −10.58 to −6.11) and diastolic blood pressure (WMD −3.82 mmHg; 95% CI −6.69 to −0.96) in hypertensive patients, while garlic did not show a significant effect on lowering blood pressure in normotensive participants.

### 3.4 Inflammatory outcome

There was only one inflammatory outcome, C‐reactive protein, reported in a meta‐analysis of nine intervention studies (Taghizadeh et al., 2018). The results of the random effects model showed that garlic significantly reduced the level of C‐reactive protein (WMD −0.80 mg/L; 95% CI −1.50 to −0.10) (Table 3).

### 3.5 Side effects

According to the reports of the included meta‐analyses (Ried, 2016; Ried, Toben, et al., 2013; Sahebkar et al., 2016; Wang et al., 2017), garlic led to a very low incidence of complications. The most common side effects were garlic odor, breath or taste, and gastrointestinal complaints such as mild discomfort, flatulence, bloating, reflux, and belching. It was suggested that the side effects mentioned above were not associated with the type of garlic preparation or dosage, and a rare incidence of allergic reactions to garlic intake was also reported. No obvious side effects were reported for other types of allium vegetables in the included meta‐analyses.

### 3.6 Heterogeneity of included outcomes

Among the 50 outcomes, the *I^2^* value was not reported for nine outcomes, and we did not conduct the *I^2^* statistic for the assessment of heterogeneity because of the lack of available data. For the other 41 outcomes, 29 had an *I* ^2^ > 50%, and the *I^2^* values of the remaining 12 outcomes were <50%. A total of 46 outcomes were analyzed with the random effects model, and only four outcomes were analyzed by using the fixed effects model. The heterogeneity in the included meta‐analyses was affected by many factors, such as the specific type and form of the allium vegetables, the dose of the allium vegetables, the health status of the participants, and the duration of each primary study. To reduce the heterogeneity, we stratified the outcomes with different allium vegetables, participants, and durations using the available data.

### 3.7 Publication bias of the included outcomes

Of the 50 outcomes, 14 had no assessment of publication bias, and we did not perform Egger's regression test for these outcomes because they contained too few studies or lacked available data. For the remaining outcomes with assessments of publication bias, 55% were assessed with Egger's regression test, 14% were assessed with Begg's test, and 31% were assessed with a funnel chart. In Egger's regression test, eight outcomes had a *p* > 0.1, seven outcomes had a *p* < 0.1, and five outcomes were reported as having no publication bias. In the funnel chart assessment, four outcomes were reported as having no publication bias, five had a low risk of publication bias, two had a moderate risk of publication bias, and no publication bias was reported in five outcomes according to Begg's test. Statistically significant publication bias was shown for lipoprotein (a) with garlic, gastric cancer with onion, colon cancer and rectal cancer with garlic, and prostate cancer with garlic and onion, and a moderate risk of publication bias was shown for HbA1c with a duration of 12 and 24 weeks.

### 3.8 Results of AMSTAR and GRADE assessment

In total, for the 16 most recent meta‐analyses with 50 outcomes, the median AMSTAR score was 7.5 (range 6–10). The median AMSTAR score was 8 (range 7–8) for meta‐analyses of cancer and tumor outcomes and 8 (range 6–9) for meta‐analyses of metabolic outcomes; the AMSTAR scores were 6 and 10 for two meta‐analyses of cardiovascular outcomes. Studies without assessments of heterogeneity or publication bias were marked with lower scores. According to the quality assessment criteria of GRADE, 26 outcomes were rated as “very low,” 18 outcomes were “low,” five outcomes were “moderate,” and only one outcome was rated as “high.” Among the outcomes with “low” or “very low” quality of evidence, the majority had lower quality because of the risk of publication bias, inconsistency, and imprecision; in particular, outcomes of meta‐analyses of observational studies were more likely to have a serious risk of bias. Detailed information about the AMSTAR and GRADE assessments is shown in Table 4.

**Table 4:** Assessments of AMSTAR and GRADE system

Outcome	Author	Year	Type of allium vegetables	Population	AMSTAR	Risk of bias	Inconsistency	Indirectness	Imprecision	Publication bias	Quality of evidence
Gastric cancer	Li, Z. Y., et al	2018	Garlic or garlic powder	Participants from Asia, Europe, and America	8	Serious	Serious	Not serious	Not serious	None	Low
Gastric cancer	Turati, F., et al	2015	Onion	Participants from Asia, Europe, and South America	7	Serious	Serious	Not serious	Serious	Yes	Very low
Gastric cancer	Turati, F., et al	2015	Chinese chives	Participants from Asia, Europe, and South America	7	Serious	Serious	Not serious	Serious	None	Very low
Gastric cancer	Turati, F., et al	2015	Allium vegetables	Participants from Asia, Europe, and South America	7	Serious	Serious	Not serious	Serious	None	Very low
Colorectal cancer	Chiavarini, M., et al	2016	Garlic or garlic supplement	Participants from China, Europe, Argentina, and America	8	Serious	Serious	Not serious	Not serious	None	Low
Colorectal cancer	Turati, F., et al	2014	Onion	Participants from Asia, Europe, Australia, and North and South America	7	Serious	Not serious	Not serious	Serious	None	Low
Colorectal cancer	Zhu, B. B., et al	2014	Allium vegetables	Participants from China, the Netherlands, and America	8	Serious	Not serious	Not serious	Serious	None	Low
Colorectal cancer of male	Chiavarini, M., et al	2016	Garlic or garlic supplement	Participants from China, Europe, Argentina, and America	8	Serious	Not serious	Not serious	Serious	None	Low
Colorectal cancer of female	Chiavarini, M., et al	2016	Garlic or garlic supplement	Participants from China, Europe, Argentina, and America	8	Serious	Serious	Not serious	Serious	Yes	Very low
Colon cancer	Chiavarini, M., et al	2016	Garlic or garlic supplement	Participants from China, Europe, Argentina, and America	8	Serious	Serious	Not serious	Serious	Yes	Very low
Rectal cancer	Chiavarini, M., et al	2016	Garlic or garlic supplement	Participants from China, Europe, Argentina, and America	8	Serious	Not serious	Not serious	Serious	Yes	Very low
Colorectal adenomatous polyps	Turati, F., et al	2014	Allium vegetables	Participants from America	8	Serious	Not serious	Not serious	Serious	None	Low
Upper aerodigestive tract cancer	Guercio, V., et al	2016	Allium vegetables	Worldwide countries	7	Serious	Serious	Not serious	Serious	None	Very low
Upper aerodigestive tract cancer	Guercio, V., et al	2016	Garlic	Worldwide countries	7	Serious	Serious	Not serious	Not serious	None	Low
Upper aerodigestive tract cancer	Guercio, V., et al	2016	Onion	Worldwide countries	7	Serious	Serious	Not serious	Serious	None	Very low
Esophagus cancer	Guercio, V., et al	2016	Allium vegetables	Worldwide countries	7	Serious	Serious	Not serious	Serious	Yes	Very low
Esophagus cancer	Guercio, V., et al	2016	Garlic	Worldwide countries	7	Serious	Serious	Not serious	Serious	Yes	Very low
Esophagus cancer	Guercio, V., et al	2016	Onion	Worldwide countries	7	Serious	Serious	Not serious	Serious	Yes	Very low
Head and neck cancer	Guercio, V., et al	2016	Garlic	Worldwide countries	7	Serious	Serious	Not serious	Serious	Yes	Very low
Head and neck cancer	Guercio, V., et al	2016	Onion	Worldwide countries	7	Serious	Serious	Not serious	Serious	Yes	Very low
Larynx cancer	Guercio, V., et al	2016	Onion	Worldwide countries	7	Serious	Serious	Not serious	Serious	Yes	Very low
Prostate cancer	Zhou, X. F., et al	2013	Garlic	Participants from Asia, Europe, Australia, and America	8	Serious	Serious	Not serious	Serious	Yes	Very low
Prostate cancer	Zhou, X. F., et al	2013	Onion	Participants from Asia, Europe, Australia, and America	8	Serious	Not serious	Not serious	Serious	Yes	Very low
Total cholesterol	Shabani, E., et al	2018	Garlic	Patients with dyslipidemia	7	Serious	Serious	Not serious	Not serious	Yes	Very low
HDL	Shabani, E., et al	2018	Garlic	Patients with dyslipidemia	7	Serious	Serious	Not serious	Not serious	Yes	Very low
LDL	Shabani, E., et al	2018	Garlic	Patients with dyslipidemia	7	Serious	Serious	Not serious	Not serious	Yes	Very low
Triglycerides	Shabani, E., et al	2018	Garlic	Patients with dyslipidemia	7	Serious	Serious	Not serious	Not serious	Yes	Very low
Total cholesterol^a^	Ried, K., et al	2013	Garlic (powder, oil, garlic extract or raw garlic)	Both healthy participants and patients with dyslipidemia	8	Not serious	Serious	Not serious	Not serious	None	Moderate
HDL^a^	Ried, K., et al	2013	Garlic (powder, oil, garlic extract or raw garlic)	Both healthy participants and patients with dyslipidemia	8	Not serious	Not serious	Not serious	Not serious	None	High
LDL^a^	Ried, K., et al	2013	Garlic (powder, oil, garlic extract or raw garlic)	Both healthy participants and patients with dyslipidemia	8	Not serious	Serious	Not serious	Not serious	None	Moderate
Triglycerides^a^	Ried, K., et al	2013	Garlic (powder, oil, garlic extract or raw garlic)	Both healthy participants and patients with dyslipidemia	8	Not serious	Serious	Not serious	Not serious	None	Moderate
Total cholesterol	Ried, K., et al	2013	Garlic (powder, oil, garlic extract or raw garlic)	Participants with short duration: 2–8 weeks	8	Not serious	Not serious	Not serious	Serious	Yes	Low
Total cholesterol	Ried, K., et al	2013	Garlic (powder, oil, garlic extract or raw garlic)	Participants with long duration: >8 weeks	8	Not serious	Serious	Not serious	Not serious	Yes	Low
Total cholesterol	Ried, K., et al	2013	Garlic (powder, oil, garlic extract or raw garlic)	Participants with TC baseline ≤200 mg/dl	8	Not serious	Not serious	Not serious	Serious	Yes	Low
Fasting blood glucose (FBG)	Shabani, E., et al	2018	Garlic	Diabetic patients	7	Serious	Serious	Not serious	Serious	None	Very low
FBG	Emami, S., et al	2017	Garlic (garlic food or supplement)	Healthy participants	6	Not serious	Not serious	Not serious	Serious	None	Moderate
HbA1c	Shabani, E., et al	2018	Garlic	Diabetic patients	7	Serious	Serious	Not serious	Serious	Yes	Very low
HbA1c	Wang, J., et al	2017	Garlic supplement	Type 2 diabetic patients with duration of 12 weeks	9	Not serious	Serious	Not serious	Serious	Yes	Very low
HbA1c	Wang, J., et al	2017	Garlic supplement	Type 2 diabetic patients with duration of 24 weeks	9	Not serious	Serious	Not serious	Serious	Yes	Very low
Serum fructosamine	Wang, J., et al	2017	Garlic supplement	Type 2 diabetic patients with duration of 1–2 weeks	9	Not serious	Serious	Not serious	Serious	None	Low
Serum fructosamine	Wang, J., et al	2017	Garlic supplement	Type 2 diabetic patients with duration of 3–4 weeks	9	Not serious	Serious	Not serious	Serious	None	Low
Lipoprotein (a)	Sahebkar, A., et al	2016	Garlic supplement	Patients with dyslipidemia	8	Not serious	Serious	Not serious	Serious	Yes	Very low
Apolipoprotein B	Zeng, T., et al	2012	Garlic	Both healthy and hypercholesterolemic participants	8	Not serious	Not serious	Not serious	Serious	None	Moderate
Systolic blood pressure	Ried, K.	2016	Garlic (powder, extract or oil)	Both normotensive and hypertensive participants	6	Not serious	Serious	Not serious	Not serious	Yes	Low
Diastolic blood pressure	Ried, K.	2016	Garlic (powder, extract or oil)	Both normotensive and hypertensive participants	6	Not serious	Serious	Not serious	Not serious	Yes	Low
Systolic blood pressure	Ried, K.	2016	Garlic (powder, extract or oil)	Hypertensive participants	6	Not serious	Not serious	Not serious	Serious	Yes	Low
Diastolic blood pressure	Rohner, A., et al	2015	Garlic (powder, extract)	Hypertensive participants	10	Not serious	Serious	Not serious	Serious	None	Low
Systolic blood pressure	Ried, K.	2016	Garlic (powder, extract or oil)	Normotensive participants	6	Not serious	Serious	Not serious	Serious	Yes	Very low
Diastolic blood pressure	Ried, K.	2016	Garlic (powder, extract or oil)	Normotensive participants	6	Not serious	Serious	Not serious	Not serious	Yes	Low
C‐reactive protein level	Taghizadeh, M., et al	2018	Garlic and garlic supplement	Healthy participants or patients of type 2 diabetes and coronary artery disease	9	Not serious	Serious	Not serious	Serious	None	Low

Note

AMSTAR, a measurement tool to assess systematic reviews; GRADE, Grading of Recommendations Assessment, Development, and Evaluation.

a Patients that requiring cholesterol‐lowering medical treatments were excluded.

## 4 DISCUSSION

In this umbrella review, by summarizing the evidence from related systematic reviews and meta‐analyses, we developed an overview of the associations between allium vegetable consumption and multiple health‐related outcomes. Evidence from observational studies indicated that allium vegetables were beneficial for the prevention of gastric, esophageal, laryngeal, and prostate cancer and colorectal adenomatous polyps. Evidence from intervention studies suggested that garlic consumption was beneficial for lowering blood pressure, lipid profiles, and glucose parameters, especially in patients with hypertension, dyslipidemia, and diabetes.

The AMSTAR criteria were applied to assess the methodological quality of the included studies, and we also used the GRADE system to assess the quality of evidence. Among the 16 most recent meta‐analyses with 50 outcomes, 65% of the meta‐analyses were rated ≥8 scores of AMSTAR, and 88% of the total outcomes were rated as “very low” and “low” based on the GRADE. We found that high AMSTAR scores of meta‐analyses did not indicate high‐quality evidence of the related outcomes. Because some of the outcomes were reported as subgroup results in the related meta‐analyses, these outcomes tended to contain fewer primary studies and participants, which might lower the quality of evidence. Outcomes with too many or too few primary studies tended to have higher values of *I* ^2^ in this umbrella review, and significant heterogeneity also decreased the GRADE classifications.

As reported in the included meta‐analyses, the most common side effects of garlic intake were garlic odor and gastrointestinal complaints, and no obvious side effects were reported for the other types of allium vegetables. Moreover, basic studies showed that aged garlic extract could suppress collagen‐induced platelet aggregation via the inhibition of the P2X1 receptor, thromboxane receptor, and MAP kinase phosphorylation by acting on newly formed platelets from bone marrow megakaryocytes (Morihara & Hino, 2017), and it was also suggested that the aged garlic extract might inhibit platelet aggregation by increasing cyclic nucleotides and inhibiting fibrinogen binding and platelet shape change (Rahman, Lowe, & Smith, 2016). Although it was suggested that the aged garlic extract might influence the function of platelets, the results from animal studies indicated that the aged garlic extract was not associated with serious hemorrhagic risk (Morihara & Hino, 2017), and a randomized placebo‐controlled study also showed that the daily consumption of 4.2 g raw garlic did not impair the platelet function of healthy participants (Scharbert, Kalb, Duris, Marschalek, & Kozek‐Langenecker, 2007). Therefore, except for patients with a foreseeable risk of bleeding, garlic could be safe for dietary consumption.

Garlic and onion were the two most important allium vegetables, and it was suggested that they share many molecular properties, such as containing flavonoid and polyphenol compounds, which could act as pro‐oxidants to induce a protective response in cells and stimulate the body's own antioxidant systems (de Giorgio & Stebbing, 2016). Mechanisms underlying how allium vegetables improve multiple health‐related outcomes are complex and not completely understood; however, some studies have identified several possible mechanisms of how garlic and onion play protective roles in health‐related outcomes.

Basic experiments have indicated that both aged garlic extract and fresh garlic extract had anticancer effects. The aged garlic extract had cytotoxic effects on cancer cells by activating K+/H+ exchange, causing oxidative stress in the mitochondria and inducing mitochondrial permeability transition (Ohkubo et al., 2018). Fresh garlic extract was suggested to suppress cancer cell proliferation, which was related to increased endoplasmic reticulum stress (Petrovic et al., 2018). S‐allylmercaptocysteine, one of the water‐soluble organosulfur garlic derivatives, was suggested to suppress the growth of gastric cancer in in vivo conditions because of its effect on antiproliferation, apoptosis induction, and the regulation of the MAPK and PI3K/Akt pathways (Zhu et al., 2017). Moreover, several compounds extracted from allium vegetables, such as allyl‐thiosulfinates, have shown inhibitory effects on the growth of *Helicobacter pylori* (Canizares et al., 2004; Sivam, Lampe, Ulness, Swanzy, & Potter, 1997). Considering that *H. pylori* infection is a risk factor for gastric cancer (Lee et al., 2016), this may partly explain why allium vegetable consumption was associated with a decreased risk of gastric cancer. In addition, in vitro experiments showed that crude garlic extract could inhibit the proliferation of human cancer cell lines, such as prostate cancer cell lines, and could also induce cell cycle arrest and apoptosis in cancer cells (Bagul, Kakumanu, & Wilson, 2015).

Interestingly, six outcomes from this umbrella review all showed that allium vegetable consumption was not significantly associated with colorectal cancer. However, we noticed that basic experiments seemed to have different results. In an in vivo study, flavonoids extracted from onion seemed to play a role in inhibiting colorectal cancer and decreasing hyperlipidemia (Jin, He, Gong, Zhang, & Zhou, 2015). In an in vitro study, 8‐C‐(E‐phenylethenyl) quercetin, a novel quercetin derivative that can form in onion/beef soup, was suggested to inhibit colon cancer cell growth by inducing autophagic cell death through ERK activation (Zhao et al., 2017). The flavonoids with anticancer effects mentioned above were consumed at high doses in the in vivo study (Jin et al., 2015), and it might be difficult to reach the anticancer doses in a normal human diet. Moreover, compared with the environment in vitro, colorectal cancer is located in the lower digestive tract in humans where the environment is more complex and contains a wide variety of microbes. Therefore, we hypothesize that the anticancer compounds in allium vegetables might be absorbed or destroyed in the gastrointestinal tract before they reach the site of colorectal cancer, which might account for why the benefits of allium vegetable consumption were shown in gastric, esophageal, and laryngeal cancer but not in colorectal cancer in this umbrella review.

Basic experiments have also studied the possible mechanisms of how allium vegetables improve metabolic, cardiovascular, and inflammatory outcomes. Animal studies confirmed that aged garlic extract could suppress the increase in plasma glycated albumin levels in spontaneous type 2 diabetes mouse models, and this effect might be partly explained by the activation of AMP‐activated protein kinase followed by the suppression of free fatty acid production and MCP1 gene expression in adipose tissue (Miki et al., 2017). In Sprague‐Dawley rats fed a high‐fat diet, oral administration of garlic and onion oil suppressed the high‐fat diet‐induced body weight gain, decreased the serum levels of TGs, TC, and LDL, and increased the level of HDL (Yang et al., 2018). However, the specific mechanism was unclear. Experiments in vitro suggested that alliin, which is a garlic organosulfur compound, could attenuate 1,3‐dichloro‐2‐propanol‐induced lipogenesis via the activation of the AMPK–SREBP signaling pathway in HepG2 cells, and this mechanism might partly explain how garlic influenced lipid metabolism (Lu et al., 2018). Moreover, in dextran sulfate sodium‐induced colitis mouse models, alliin ameliorated ulcerative colitis. Moreover, in in vitro lipopolysaccharide‐stimulated RAW264.7 cell models, alliin also inhibited the inflammatory response, and this effect might be explained by alliin suppressing the MAPK‐PPAR‐γ/NF‐κB/AP‐1/STAT‐1 signaling pathways and ameliorating gut inflammation (Shi et al., 2017).

Although this umbrella review included multiple health‐related outcomes, there were also some other health‐related outcomes that have been reported in individual studies instead of meta‐analyses. Regarding serum inflammatory markers, in addition to the C‐reactive protein included in this umbrella review, a randomized clinical trial also suggested that the administration of 400 mg of standardized garlic extract twice a day for 8 weeks resulted in a significant reduction in interleukin‐6 and erythrocyte sedimentation rate (Zare, Alirezaei, Bakhtiyari, & Mansouri, 2019). Another randomized clinical trial reported that daily consumption of aged garlic extract improved oral health by reducing gingival inflammation and gingival bleeding compared to the effects observed in the placebo control (Zini, Mann, Mazor, & Vered, 2018). Black garlic, which is made from fresh garlic under high temperatures and humidity, might be helpful for patients with coronary heart disease because it increases antioxidant levels and improves the scores of quality of life, brain natriuretic peptide precursor N‐terminal, and left ventricular ejection fraction (Liu, Zhang, Cong, & Wen, 2018). These findings further demonstrated the broad health benefits of garlic consumption.

There were several strengths in this study. We first conducted this umbrella review to investigate the associations between the consumption of several types of allium vegetables and multiple health‐related outcomes. We systematically searched the relevant meta‐analyses and developed an overview of this issue. To show more specific information, we stratified the same health‐related outcomes by different factors, such as type of allium vegetables, populations, and durations. We found that even for the same outcomes with the consumption of the same type of allium vegetables, the results varied in different populations. For instance, the consumption of garlic significantly lowered the blood pressure of hypertensive participants but not normotensive participants, and the consumption of garlic was associated with decreased FBG in diabetic patients but not in healthy participants. We also summarized all the possible side effects of garlic consumption, and there was no evidence suggesting serious side effects in the current studies.

There were also some limitations in this umbrella review. The forms of allium vegetable consumption were different among the included studies. For example, the forms of garlic consumption included garlic powder, garlic oil, raw garlic, garlic extract, and other garlic supplements. Different garlic forms may lead to small differences in the effects on multiple health‐related outcomes, and we did not perform subgroup analysis for garlic forms separately because of the lack of available data. Although we found that longer durations of garlic consumption might result in more significant decreases in serum TC, HbA1c, and fructosamine levels, it remains unclear whether longer durations of garlic consumption could be more beneficial for other outcomes and how long the most appropriate duration of garlic consumption is. In addition, no evidence has suggested the appropriate dose range of daily allium vegetable consumption. Further well‐designed interventional and cohort studies are needed to address these limitations in the future.

In conclusion, allium vegetables, as common vegetables consumed around the world, were suggested to be beneficial for cancer prevention in this umbrella review. In particular, garlic seemed to be comparatively safe and recommended as a long‐term dietary component for patients with dyslipidemia, diabetes, and hypertension.

## CONFLICT OF INTEREST

None.

## AUTHOR CONTRIBUTIONS

QYW and NL contributed equally to this study. QYW and YZ designed the study; QYW and NL analyzed data; QYW, RZ, MSY, QSX, and LD wrote the paper; YZ had primary responsibility for final content. All authors read and approved the final manuscript.

## ETHICAL STATEMENT

This study does not involve any human or animal testing.
