# Supplementary material for: Allium vegetable consumption and health: An umbrella review of meta‐analyses of multiple health outcomes
Source: Food Sci Nutr. 2019 Jul 10;7(8):2451–70. doi: 10.1002/fsn3.1117 (PMC6694434; doi:10.1002/fsn3.1117)

Search strategies：

PubMed:

History

Download history Clear history

Search	Add to builder	Query	Items found	Time
#3	Add	Search (((meta-analys*) OR systematic review)) AND ((((((allium*) OR garlic*) OR onion*) OR shallot*) OR leek*) OR chive*)	18704	21:29:21
#2	Add	Search (meta-analys*) OR systematic review	2342840	21:28:29
#1	Add	Search (((((allium*) OR garlic*) OR onion*) OR shallot*) OR leek*) OR chive*	35307	21:27:53

Web of Science:

Set	Results	Save History / Open Saved History	Combine Sets (AND / OR) Combine	Delete Sets (Select All / Delete)
# 9	107	#8 AND #7<br>*Databases= WOS, DIIDW, INSPEC, KJD, MEDLINE, RSCI, SCIELO Timespan=All years*<br>*Search language=Auto*		
# 8	110,676	#6 OR #4 OR #3 OR #2 OR #1<br>*Databases= WOS, DIIDW, INSPEC, KJD, MEDLINE, RSCI, SCIELO Timespan=All years*<br>*Search language=Auto*		
# 7	191,048	TOPIC: (meta-analys*)<br>*Databases= WOS, DIIDW, INSPEC, KJD, MEDLINE, RSCI, SCIELO Timespan=All years*<br>*Search language=Auto*		
# 6	2,513	TOPIC: (chive*)<br>*Databases= WOS, DIIDW, INSPEC, KJD, MEDLINE, RSCI, SCIELO Timespan=All years*<br>*Search language=Auto*		
# 5	7,481	TOPIC: (leek*)<br>*Databases= WOS, DIIDW, INSPEC, KJD, MEDLINE, RSCI, SCIELO Timespan=All years*<br>*Search language=Auto*		
# 4	3,647	TOPIC: (shallot*)<br>*Databases= WOS, DIIDW, INSPEC, KJD, MEDLINE, RSCI, SCIELO Timespan=All years*<br>*Search language=Auto*		
# 3	60,390	TOPIC: (onion*)<br>*Databases= WOS, DIIDW, INSPEC, KJD, MEDLINE, RSCI, SCIELO Timespan=All years*<br>*Search language=Auto*		
# 2	49,727	TOPIC: (garlic*)<br>*Databases= WOS, DIIDW, INSPEC, KJD, MEDLINE, RSCI, SCIELO Timespan=All years*<br>*Search language=Auto*		
# 1	28,179	TOPIC: (allium*)<br>*Databases= WOS, DIIDW, INSPEC, KJD, MEDLINE, RSCI, SCIELO Timespan=All years*<br>*Search language=Auto*		

Embase:

▼ **Search History** (3) View Saved

# ▲	Searches	Results	Type	Actions	Annotations
1	(allium* or garlic* or onion* or chive* or shallot* or leek*).af.	24013	Advanced	Display Results | More ▾	
2	(systematic review or meta-analys*).af.	376909	Advanced	Display Results | More ▾	
3	1 and 2	432	Advanced	Display Results | More ▾	

Save Remove Combine with: AND OR

Save All Edit Create RSS View Saved

Cochrane Library：

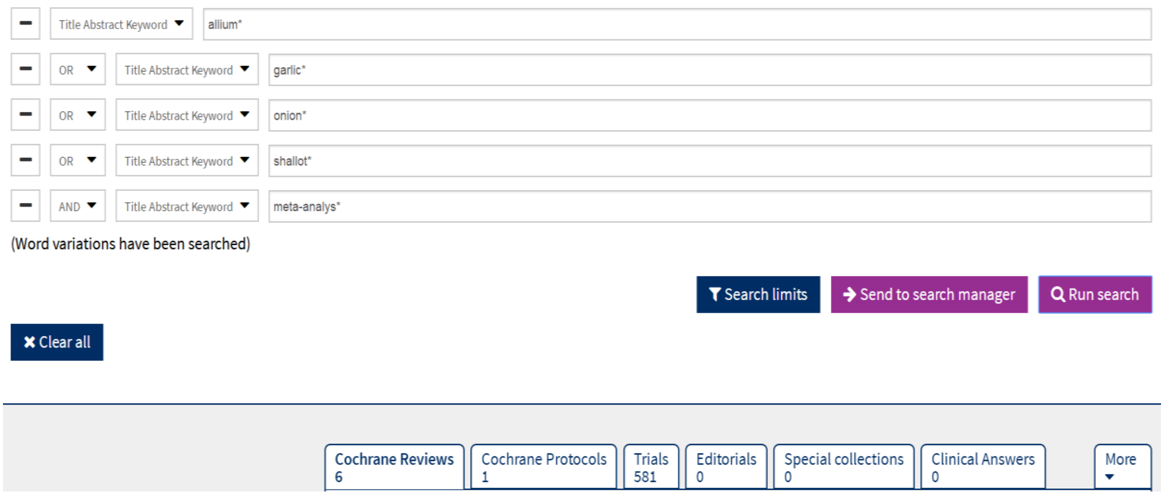

Supplement: Supplementary file 1 [file FSN3-7-2451-s001.pdf]
